# Recycling of Reinforced Glass Fibers Waste: Current Status

**DOI:** 10.3390/ma15041596

**Published:** 2022-02-21

**Authors:** R. M. Gonçalves, Alberto Martinho, J. P. Oliveira

**Affiliations:** UNIDEMI, Department of Mechanical and Industrial Engineering, NOVA School of Science and Technology, Universidade NOVA de Lisboa, 2829-516 Caparica, Portugal; ajmm@fct.unl.pt

**Keywords:** recycling, composites, reinforced glass fibers, waste management

## Abstract

In this paper, a review of the current status and future perspectives for reinforced glass fiber waste is undertaken, as well as an evaluation of the management hierarchy for these end-of-life materials. Waste levels are expected to increase in the coming years, but an improvement of collection routes is still necessary. The recycling processes for these materials are presented. The associated advantages and disadvantages, as well as the corresponding mechanical characteristics, are described. Although mechanical shredding is currently the most used process, there is a potential for thermal processes to be more competitive than others due to the fiber quality after the recycling process. However, the energy requirements of each of the processes are not yet well explained, which compromises the determination of the economic value of the recycled fibers when included in other products, as well as the process feasibility. Nevertheless, the work of some authors that successfully integrated recycled glass fibers into other elements with increased mechanical properties is evaluated. Future recommendations for the recycling of glass fiber and its commercialization are made.

## 1. Introduction

The use of composite materials is more present than ever in our day-to-day life [1]. Composite materials consist of two or more materials, to form a new one with characteristics that are a mix of those exhibited by the constituent materials. Generally, composite materials are based on two distinct phases: a matrix and a reinforcement.

Polymer matrix composites are the most used materials in common applications due to their versatility and easy production routes. The reinforcement is commonly stronger and stiffer than the matrix, with fibers, either continuous or discontinuous, being the most used option [2,3]. The matrix often consists of thermoplastic or thermoset resin, which acts as a binder by surrounding the fiber mat or roving. Furthermore, composite materials often have foam cores to reduce weight and cost or metal inserts to facilitate assembly into other components [3,4]. In fact, the mechanical properties of the fibers (with tensile strength ranging from 2415 to 4890 MPa [5]), associated with a relative low density (2.11 to 2.72 g/cm^3^ [6]), combined with the low tensile strength (27 to 137 MPa [7]), low density (0.98 to 1.2 g/cm^3^ [8]), and excellent thermal and electrical insulating attributes of thermoset polymers, can provide outstanding properties.

Composite materials are desirable for design engineers in the construction sector to build elaborate and irregular envelopes and facades [9]. In the wind energy sector, as turbine blades become longer than 50 m, the use of polymer matrix composites is the most sought-after solution [10]. The automotive industry also uses composite materials to produce sheet molding compound (SMC) and dough molding compound (DMC), as well as boat hulls [1,11]. In the aeronautic sector, with increasingly demanding flight distance and CO_2_ reduction targets [12], the use of composites is of increased importance. These sectors require composite materials, especially glass and carbon fiber-reinforced polymer (FRP), due to its high strength, lightness, and corrosion resistance over time [2]. 

Despite all the advantages mentioned above, the great handicap when using a polymer matrix composite is the matrix nature. Unlike thermoplastic polymers which can be easily remolded and reused, thermoset polymers with the most common resins such as epoxy, polyester, and vinyl ester are not easily depolymerized from their original constituents, creating a significant challenging for end-of-life structures now and in the near future [4,13]. 

Thermoset polymers constitute around 70% (in weight) of the resins used as a matrix in FRP composites. In the case of glass fiber-reinforced polymers (GFRPs), practically all products on the market use a thermoset plastic as a matrix. These polymers present better mechanical properties, when compared with those of a thermoplastic nature, and greater resistance to temperature, gas penetration, and impact [9].

### The Need to Recycle: Market Size and Motivations

Different markets are increasingly looking toward using composite materials, while well-established sectors are growing in terms of their usage [14]. Inevitably, the result will be more waste; thus, there is a strong need to evaluate recycling strategies for these materials.

According to the European Composites Industry Association (EuCia), GFRP represents 95% of all FRP composites, and its use is expected to steadily grow by 5.6% annually, although the COVID-19 pandemic severely affected the production rate in 2020 [15]. The European GFRP market has experienced the biggest slump since the economic and financial crises of 2008/20009, with production falling 12.7% to 996,000 tons [16]. However, with economic and industrial uptake, it is expected that production will again increase, hitting the previous production quotas in less than 1 year.

**Figure 1:**
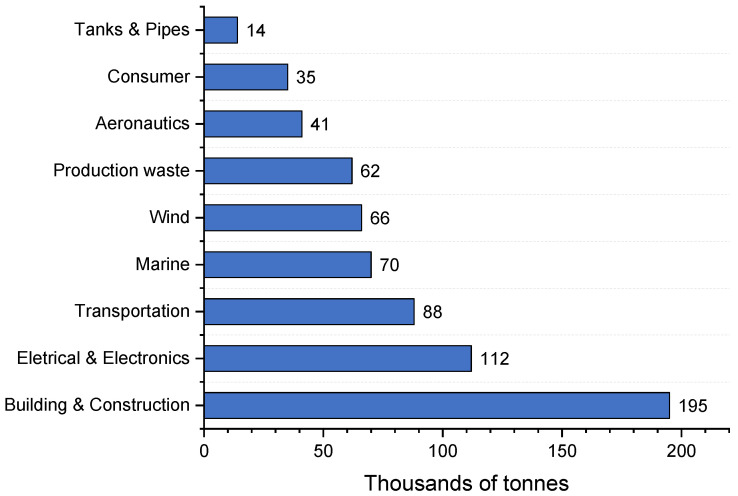
Estimated composite waste per sector in thousands of tons in 2025 (adapted from [18]).

Despite the production regression, the amount of GFRP to be recycled is expected to constantly increase [17]. Figure 1 depicts the composite waste prediction per sector for 2025 in Europe [18]. The building and construction sectors account for the biggest waste producers, followed by the electrical and electronics sector, and then the transportation industry, which includes the SMC and DMC previously described. In the automotive industry, SMC and DMC are desirable for weight reduction and moldability, with panels being produced using 50% to 70% (in weight) glass fiber [3]. There is a market cap residue of SMC and DMC materials of around nine million tons per year [19].

SMC and DMC were not considered an environmental problem, with the consequence of slowing down the development of recycling technologies. However, with increasing disposal fees and stricter legislation, particularly for the automotive industry, there has been a dramatic increase in the interest to recycle SMC and DMC over the past few years [3,20].

Another sector that has raised concerns in the scientific community due to residue predictions over time is wind energy, with an estimated composite material usage of 2.5 million tons annually [18]. Therefore, the need for a joint effort across different industries to determine suitable recycling routes for these composite materials is clear, aimed at decreasing the associated carbon footprint.

Over the last few decades, the wind energy sector has become one of the most promising sources of renewable energy, with thousands of towers being installed annually. However, the lifespan of the main structures (blades) is relatively low, becoming a problem when decommissioned. A blade’s lifecycle is around 20 to 25 years, being responsible for only 10% to 15% of the wind tower that is not fully recycled due to its composition (62% GFRP and 17% Carbon Fiber-Reinforced Polymer (CFRP), in weight). Therefore, a significant amount of material needs to be processed [21,22,23].

Projections made by EuCia indicate that around 66,000 tons of thermoset composite will originate from wind turbine blades in 2025, representing only 10% of the total estimated thermoset composite waste for the same year [24]. Looking forward to the future, there are a massive 43 million tons of blades expected to be recycled in 2050, as a consequence of the intensive installation of wind towers to fulfil green energy source targets [10,18]. Consequently, wind energy as a green source should be reconsidered.

The quantity of GFRP will inevitably increase over time. Therefore, recycling technologies will become more mature as legislation becomes more restrictive within the end-of-life exportation and landfill of composite materials. Therefore, there are two possible approaches: either science continues to develop methods to separate the reinforcement phase from the matrix or a distinct approach encompassing recycling of composite materials by processing the matrix and the reinforcement together must be adopted. Regardless of the choice, FRP will have to be consistently and successfully recycled in the near future.

In this review, an overview of the undergoing recycling technologies for GFRP waste is made on the basis of the usefulness of the retrieved fibers. We provide a brief description of the recycled material and the processes. This content is mainly obtained from the peer-reviewed literature, as well as governmental projects and company reports [4,14,17,18,23,24,25,26]. The numerical information shown throughout the paper represents typical range values of the processed materials.

## 2. Management of Glass Fiber-Reinforced Polymers Waste

Materials containing glass fiber are present in almost all materials of our society. For this reason, current and future waste management policies and environmental legislation require all engineering materials to be properly recovered and recycled [11]. This problem should be addressed not only in end-of-life products, but also in the prevention and smart usage of these materials. 

**Figure 2:**
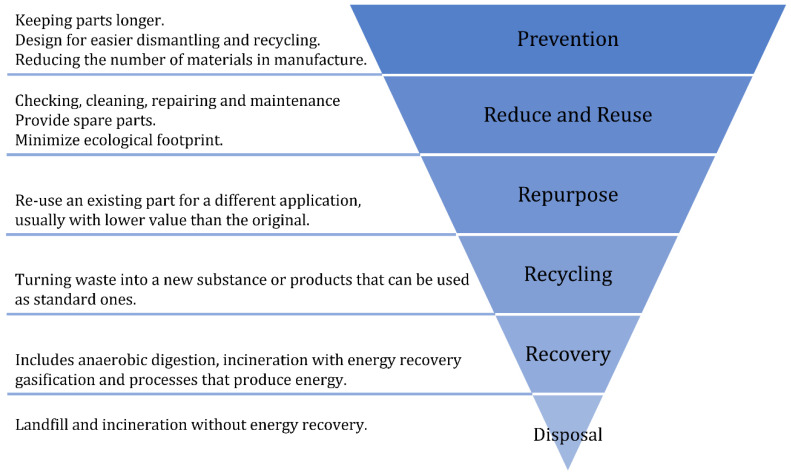
The waste management hierarchy (adapted from [31]).

GFRP waste tracking is not well established throughout the industries, resulting in the misguidance of materials from the respective recycling routes [27]. It is suspected that several tons of material never reach the appropriate recycling channels due to lack of investment and resources. For that reason, the European Union (EU) has implemented the 2008/98/EC directive [28]. This directive provides legislation, combining taxes and economic incentives, and suggests a waste management hierarchy (see Figure 2) to channel these materials into the respective recycle routes, reducing both landfill and environment impacts [29].

The 2008/98/EC directive is dedicated to the waste hierarchy, suggesting that there is no ban on landfills, but rather a definition of how the state members should address the management of specific streams of waste to reduce the impact of landfill disposal on the environment. In other words, it is a request for policymakers to make reasonable choices for product lifecycles, considering the economic viability and technical feasibility. Nevertheless, these directives have a direct impact on how each country treats its waste. For example, Germany and the United Kingdom no longer allow the landfill of GFRP [14,30]. Nonetheless, it should be emphasized that, in special circumstances, this landfill prohibition can be circumvented in these countries.

The need to recycle is with the objective of not only reducing the negative impact on the environment but also minimizing disposal costs and reducing the amount of raw materials produced. According to Glass Fiber Europe, the amount of energy required to produce glass fibers and the resin used in the matrix is 13–45 MJ/kg and 76–137 MJ/kg, respectively, with added fossil-fuel consumption and associated high environmental impacts [32,33]. Although the energy requirements of chemical and thermal recycling processes are still relatively high, with energy consumptions in the range 25–30 MJ/kg and 20–90 MJ/kg, they can still be more competitive than the production of virgin materials, allowing for the circularity of the material in a closed loop [14,34,35]. 

GFRP producers and suppliers have additional costs with their end-of-life products when sending the material to be incinerated or land-filled [13]. Thus, the European Union has implemented targets aimed at banning the exportation and landfill of thermoset composites by 2025, pushing forward the need to recycle these materials. In a world where natural elements are finite, it is of extreme important to reuse materials as much as possible, thus minimizing the production of virgin materials [36].

## 3. Fiber-Reinforced Polymer Recycling Processes

**Figure 3:**
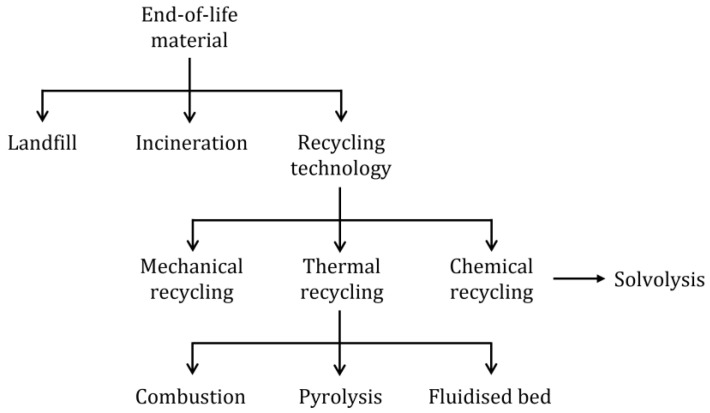
Representation of different recycling processes for thermoset composite materials.

When repurposing is not possible, recycling and recovery are the next option. Recycling implies that energy must be consumed to turn waste into something usable. Recovery requires turning waste into fuel or using it in processes that need thermal energy [37]. Figure 3 depicts a breakdown of different recycling technologies, and Table 1 summarizes the key features of different processes technologies.

### 3.1. Mechanical Recycling

**Figure 4:**
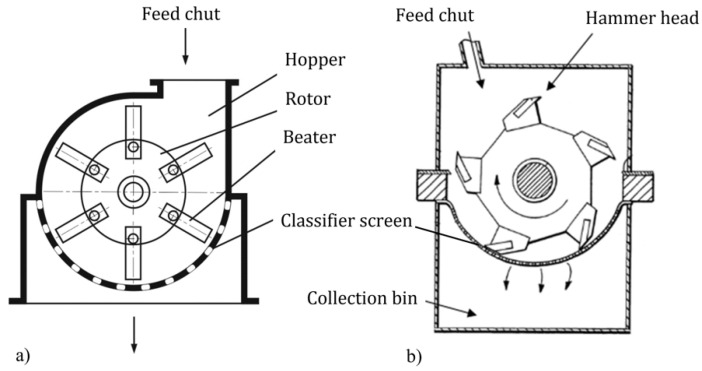
Schematics of a rotating (**a**) hammer mill and (**b**) cutting mill, with changeable classifier screen (adapted from [3]).

**Figure 5:**
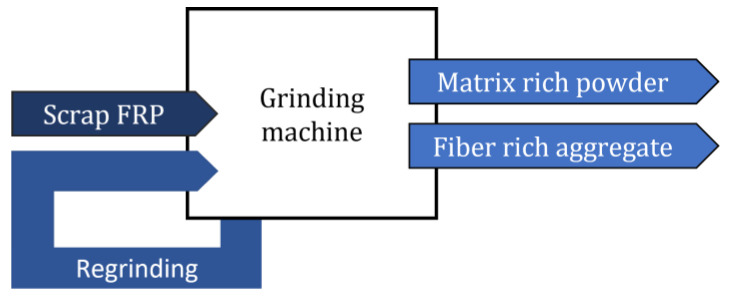
Diagram of the mechanical recycling process.

Mechanical recycling of end-of-life products is the most common process used for recycling thermoset polymers [11,13]. This process starts with a size reduction into smaller piecesm making it easier to remove metal inserts while making transportation simpler. The material is then slowly crushed (~200 rpm [52]) in a milling or grinding machine into smaller pieces [4]. Some equipment, as depicted in Figure 4, has interchangeable sieving grids integrated before the collecting bin, allowing separation of the crushed material into different sizes [1,13]. The hammer-mill type granulator is commonly the best processing equipment for the production of ready-to-use material in new composites [3]. A schematic of the process is depicted in Figure 5.

Some machines may not have a classifier screen, since they correspond to modifications of other fields such as agricultural engineering. Therefore, an additional process for classifying the material into different granulometries is necessary [38,53].

**Figure 6:**
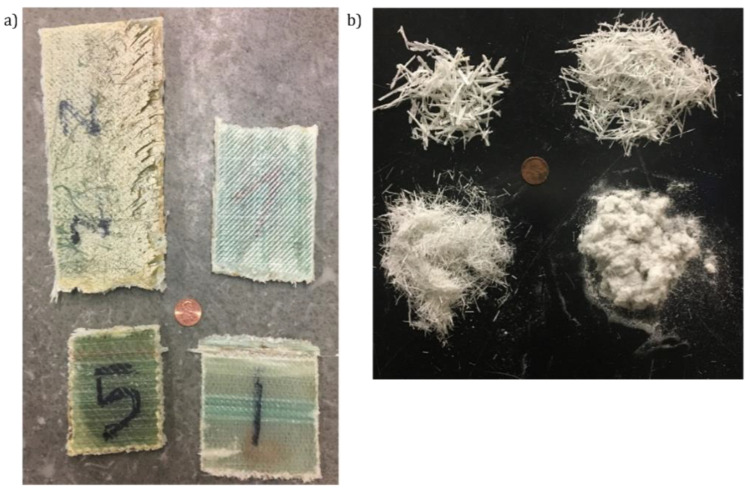
(**a**) GFRP panels; (**b**) mechanically recycled material further sieved using a shake table [54].

The result of mechanical recycling is a mixture of resin, filler (if present), and fibers with different aspect ratios that may have to be further sieved. The granulometry selection is usually done with a zig-zag classifier, with a shake table, or with a rotary sieving pan (refer to Figure 6) [1,3,25,52]. The zig-zag classifier is also known as cascade air classification, with the advantage of de-dusting the material, and it depends mainly on the air velocity. The downside of this method is that it requires an expensive technological setup.

The main advantages of mechanical recycling are industrial scalability, economic viability of the process, and low environmental impact [1,52]. However, there are also associated disadvantages. it cannot retrieve the base material in its full shape as it becomes fragmented [36], and there is a devaluation of the fiber mechanical properties with a decrease in strength of approximately 22% [3,11,14].

Despite mechanical recycling being the most used process, there are still key aspects that need development. For example, the recycled fiber strength is unclear [3]. The effect of the sieving grid geometry on the fiber aspect ratio needs to be fundamentally determined. The effect of cutting speed on material strength and matrix removal must also be clarified [4].

### 3.2. Thermal Processes

Thermal recycling is usually based on the application of heat to separate the fiber reinforcement from the matrix, with potential to be reused and with the possibility of having greater added value, particularly if it consists of carbon fibers [30].

#### 3.2.1. Combustion

This technique is characterized by the integration of the material to be recovered during the processing of another material, meaning that recycling occurs through co-processing [37]. In fact, the combustion technique does not involve material recovery, but energy recovery [11].

**Figure 7:**
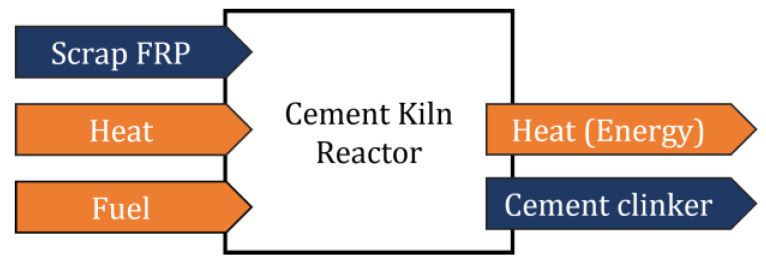
Diagram of the cement kiln process.

Combustion is mainly used via the cement kiln route where the polymer matrix is burned as fuel for the process, reducing the use of fossil fuel; the fibers containing E-Glass, which are based on alumina borosilicate, provide mineral feedstock to be used as part of the cement clinker (initial stage of the cement), with specific amounts of silica (Si), calcium (Ca), and alumina (Al) [1,55]. Further down the process, the clinker is ground to form cement. A schematic of the process is depicted in Figure 7.

The use of both GFRP and CFRP in processes with energy recovery has its advantages. Co-processing FRP through a cement kiln not only reduces the carbon footprint by up to 16% but also provides valuable materials to the process [41]. Moreover, there is full compliance with the European Waste Framework Directive (WFD) 2008/98/EC, and it is the go-to process for recycling wind turbine blades [4]. However, the following disadvantages also exist: the material organic fraction only accounts for 30% to 40% in weight, and the presence of boron in high quantities affects the cement setting behavior [14]. Furthermore, the scrap material must meet the cement kiln requirements, i.e., the inexistence of foreign materials (metal inserts or fasteners) and the need for the scrap to be smaller than a predetermined size with a specific calorific value higher than 5000 kcal/kg [1,4].

#### 3.2.2. Pyrolysis

Pyrolysis is a destructive process that converts organic material in an inert atmosphere at relative low temperatures, usually from 450 to 800 °C [20]. The temperature at which the process is completed depends on the nature of the composite matrix. Typically, polyester resins need a lower temperature, while epoxy or high-performance thermoplastics, such as PEEK, need higher temperatures to be degraded [14,42].

**Figure 8:**
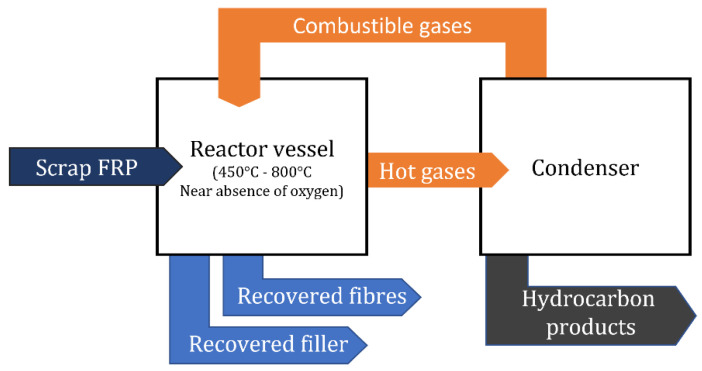
Diagram of the pyrolysis process.

This technique allows for recovery of the composite constituents with volatilization of the resin matrix into gas and oil, while the fibers and fillers (if they are present) are isolated [46]. Gas can be used as fuel for the pyrolysis system, while oil may be used as liquid fuel or returned to the refinery to be further processed to extract the polymer monomers and transform them into a new resin [2,26,55]. A schematic of the process is depicted in Figure 8.

As a result of the temperature at which the process is conducted, Oliveux et al. [26] observed that the mechanical properties of the fiber are reduced by 50% to 64% when compared to virgin ones, compromising the usefulness of the fibers as completely new ones [46]. Thus, the fibers can be used as filler or replacement in the production of new composite materials such as SMC and DMC or integrated into new polymer concretes or mortars [1,13]. Nevertheless, Yang et al. [48] studied the feasibility of regenerating thermally degraded glass fiber performance to enable the closed-loop recyclability of thermoset composite materials. The process is achieved using two chemical techniques known as chemical etching and post-salinization, successfully resulting in the regeneration of recycled fiber strength.

**Figure 9:**
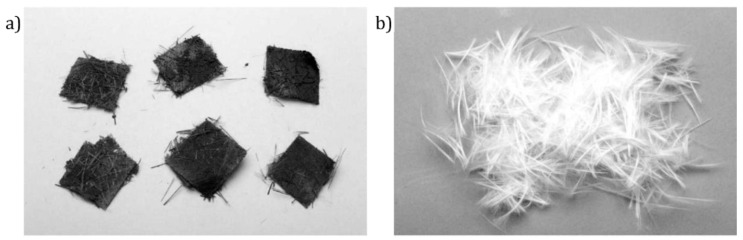
(**a**) Solid pyrolysis residues; (**b**) recovered fibers after char removal (adapted from [2]).

Pyrolysis has the advantage of not using chemical agents, the possibility of retrieving hydrocarbon products generated during the process, and low CO_2_ production [56]. However, it is typically conducted in a near absence of oxygen, resulting in char formation and fiber contamination, as can be depicted in Figure 9a [2]. The char removal process requires post-treatment in a 450 °C furnace, further damaging the fibers [2,26].

#### 3.2.3. Fluidized Bed

**Figure 10:**
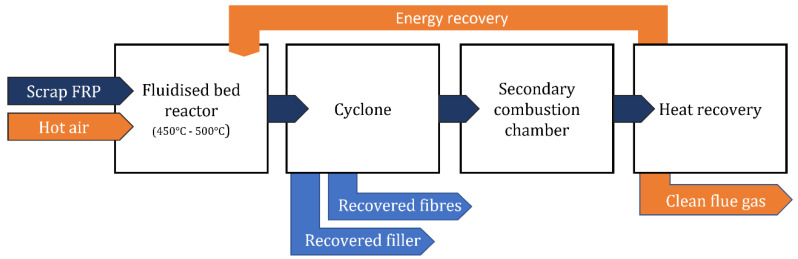
Diagram of the fluidized bed process.

Fluidization is a technology where a bed of solid particles, such as silica sand, is transformed into a fluid state through its suspension in a hot stream of air at temperatures in the range of 450 to 550 °C and streamed at speeds between 0.4 and 1.0 m/s, thus being broken into smaller pieces of FRP material [17,49]. In the chamber where FRP polymer decomposes, any existent mineral fillers or paint is removed from the fibers. The particles are elutriated from the fluidized bed with the gas stream, and the fibers are sieved in a gas–solid separation device [4,51]. Inorganic solids such as metal inserts or fasteners sink in the chamber and can be removed via a bed regrading process [4]. Furthermore, energy can be recovered from the polymer oxidation in a secondary higher-temperature combustion chamber [50]. A schematic of the process is depicted in Figure 10.

**Figure 11:**
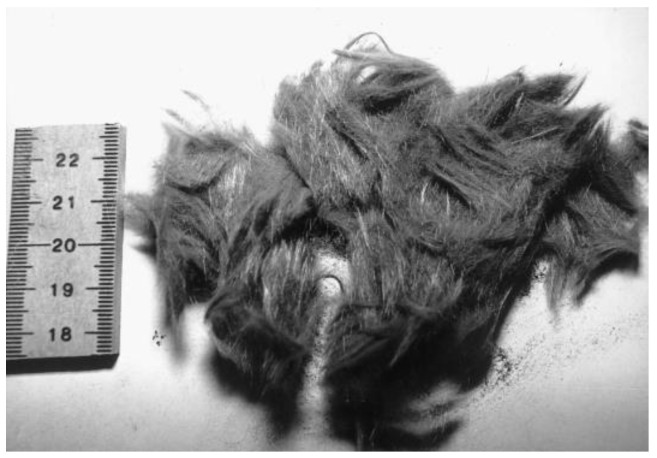
Glass fiber recovered from the fluidized bed process, [51].

Unlike pyrolysis, the fluidized bed process occurs in a rich and high-flow oxygen atmosphere, resulting in clean fibers, with very little char surface contamination, suitable to be reused in new designs, as can be seen in Figure 11. Nonetheless the reduction in fiber mechanical properties occurs due to several factors, including sand abrasion and the effects of oxidation and temperature [3]. A significant advantage of the fluidized bed process is that it is very tolerant to process-contaminated end-of-life FRP materials, unlike the cement kiln and pyrolysis routes, which do not allow for contaminated material to be properly recycled [51].

The fluidized bed process has proven its value, with recycled fibers having typically the same stiffness and a reduction in strength of approximately 50% to 75% compared to virgin E-Glass fibers, the same percentage as pyrolysis [3,49]. In addition to the thermal effect on the fibers, the fluidized bed sand agitation not only damages the fiber surface but also has annealing effects, causing stress relaxation at high temperatures [50].

Efforts to minimize the inherent disadvantages of this process were studied by Kennerley et al. [19] using a silane coating. However, it was shown that, in spite of the recoating, no significant improvements over untreated fibers were observed when used as reinforcement in new DMC formulations [3,19].

### 3.3. Chemical Recycling

**Figure 12:**
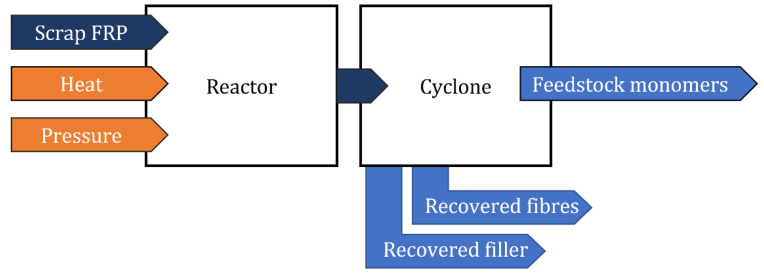
Diagram of the solvolysis process.

Chemical recycling, commonly referred to as solvolysis, involves depolymerization of the matrix with liberation of the fibers. Through the action of chemical agents, the polymer is transformed into basic monomers that can be used as feedstock or fuel to other processes or to manufacture new polymers [36,44]. Depending on the nature of the solvent, the solvolysis can be called hydrolysis (using water), glycolysis (using glycol: methanol, ethanol, 1-propanol, and acetone), or acid digestion (using acids: nitric) [43,56]. This process can be used for GFRP, as exemplified in the works of Oliveux et al. [44] and Morin et al. [56], with a focus on the chemical recyclability of SMC composites [11]. A schematic of the process is depicted in Figure 12.

Unlike the other solvolysis processes, the acid digestion route can be conducted at atmospheric pressure, which results in less expensive equipment. However, the process presents a huge demand for the use of alkaline and catalyst products, which not only are environmentally hazardous but also result in fibers with a high state of degradation and, therefore, with low commercial value [14,36]. Recently, Dang et al. [57] investigated the feasibility of decomposing epoxy resin at relatively low temperatures (T < 100 °C) using a nitric acid solution, prior to a curing process with diamino diphenyl methane. This resulted in the separation and recovery of the glass fiber from the epoxy resin. This pre-processing approach presented by Dang et al. suggests the need to implement extra steps in the recycling process rather than using more demanding processing conditions.

**Figure 13:**
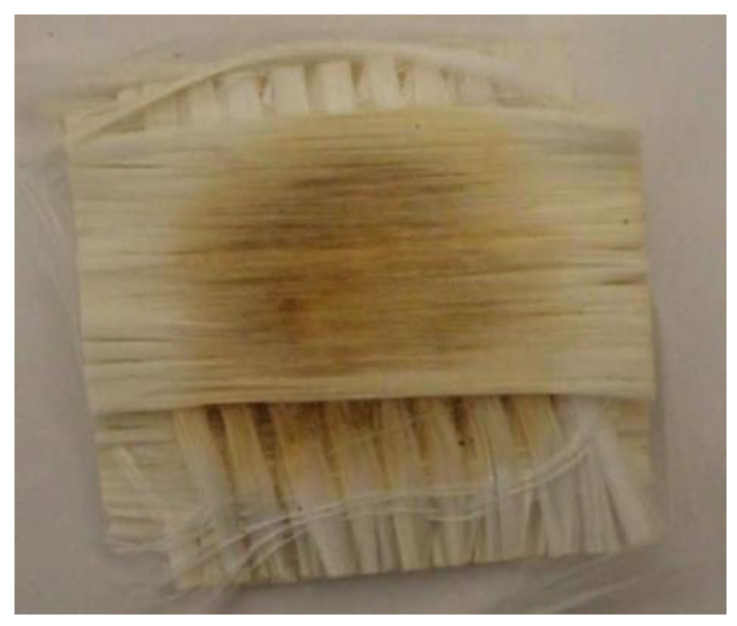
Sample (50 × 50 mm) after hydrolysis at a temperature around 300 °C [26].

The advantage of using chemical processes, especially those using water and alcohol in a near-supercritical state (T > 300 °C and P > 221 bars), is that monomers can be separated from the dissolved solution using evaporation for water and distillation for alcohol [11,44]. An experiment conducted by EURECOMP using a 20 L reactor vessel (resulting material depicted in Figure 13) involved a piece of glass fiber being recycled through solvolysis at T ≈ 300 °C and P ≈ 250 bar [58]. Diffusion problems due to the piece size can be seen in the central region. For this reason, the scalability of the process is not only dependent on the material size but also on the capability of the supercritical fluid to transport mass and diffuse through the material. Moreover, if the resin concentration is too high, the fluid gets saturated and the reaction slows down considerably [26,44]. Nevertheless, chemical processes are characterized by the capability of retrieving high-strength fibers when compared to other processes [56].

Chemical recycling requires expensive laboratory equipment, such as stainless-steel reactors, to avoid corrosion from the chemical reaction. Furthermore, a supercritical state generates considerable amounts of acid and salt waste [44]. The solvents and catalysts used have restricted disposal regulations associated with high costs and potential health and environmental impact [14]. Moreover, a large amount of heat is used over a long period of time to process the material [3]. For these reasons, chemical recycling is yet to evolve and become commercially and environmentally viable.

Despite reports indicating the viability of chemical recycling to be higher in CFRP because of the economic value of carbon fibers, there is still a chance for GFRP to be successfully recycled this way [44]. Improvements in the process should be addressed, such as milder solvolysis conditions, i.e., lower temperature and pressure, with less aggressive and more environmentally friendly solvents.

In the search for new hybrid recycling processes, Zabihi et al. [59] successfully managed to decompose the C–N bonds existent in the matrix resin, by submerging the sample in hydrogen peroxide (H_2_O_2_) and applying microwave radiation. This approach may be attractive to the scientific community as it does not require significant processing time (approximately 1 h), and the temperatures to which the materials are subjected to are relatively low (70 °C). Furthermore, the mechanical properties are very appealing as the tensile strength is only reduced by 7.3%.

### 3.4. Energy Demand and Economic Viability of Composite Recycling Methods

The impact of end-of-life materials is characterized not just by their environmental impact when decommissioned but also by the energy per weight required to be recycled or transformed into another product with added value [14]. Effective use of energy sources increases the economic potential of the process. Therefore, the appropriate recycling technologies should have a tradeoff between the consumed energy in the process and the quality and value of the recycled material [25].

**Figure 14:**
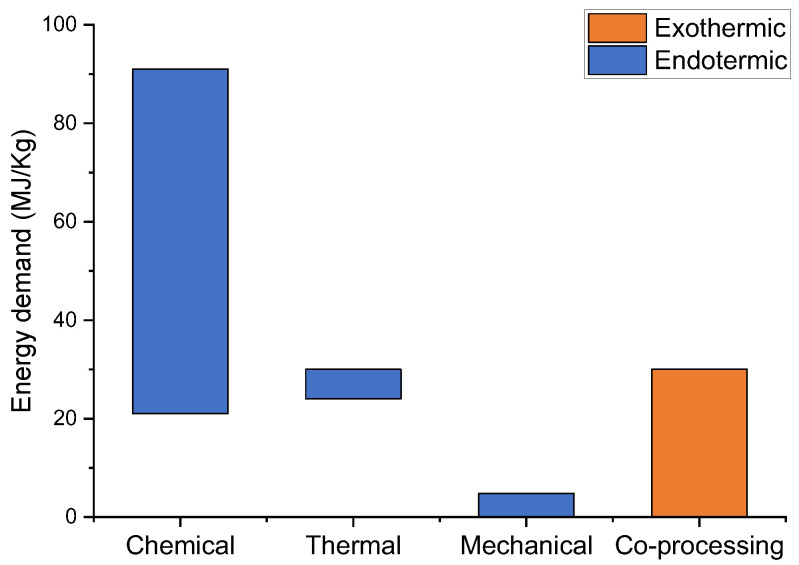
Energy demand in composite recycling technology methods (adapted from [14,34,35]).

There is no clear specific information on the GFRP recycling energy demand; thus, general information on FRP is depicted in Figure 14. Depending on the type of recycling methodology used, it is expected that the energy consumption of recycled GFRP (rGFRP) will range within the values depicted in Figure 14 [14]. Nevertheless, mechanical recycling is indeed the process that consumes the least energy [60]. This knowledge gap needs to be addressed since process energy demands can have a direct link to the product quality and feasibility of the process [14,35,52].

The viability of recycling technologies is based not only on the energy demand but also on the fiber quality and, therefore, its associated value. In any material processing route, i.e., in manufacturing, recycling, and/or remanufacturing, the energy demand dominates the environmental burden and global warming potential of the process [52]. These factors influence the final price of the recycled material, which, in the case of mechanical recycled fibers, is about 80% of virgin ones [61].

**Figure 15:**
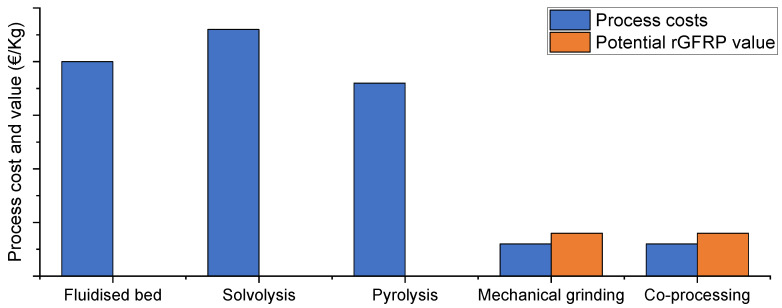
Estimated relative costs and values of composite recycling technologies (adapted from [14,18]).

Figure 15 shows the relative cost and value (EUR/kg) of different recycling processes. The size of the bars is quantitative and varies among different European Union recyclers using the same process due to varying process parameters such as throughput rates, capacity, temperature, and pressure [18,24]. No values for the thermal and chemical rGFRP value were found.

Mechanical shredding and co-processing are the least expensive processes available to recycle GFRP, with estimated costs of 150 to 300 EUR and a gate fee of approximately 150 EUR. However, due to some exceptions in country legislation, incineration and landfill are still preferred to mechanical shredding or co-processing.

Legislations in the United Kingdom and Netherlands, for example, state that. if the cost of alternative waste management routes is higher than 200 EUR/t, then landfill is allowed. According to a WindEurope survey, the cost of mechanical recycling wind turbine blades including in site pre-cut, transport, and processing is established between 500 and 1000 EUR [18]. For this reason, GFRP ends up being landfilled rather than recycled. Furthermore, a typical cement kiln route can only process approximately 15,000 tons of composite materials, which is not enough for the existing demand [24]. Looking into the remaining alternatives, the thermal processes would only be a commercially viable solution if there were more than 10,000 tons per year reaching the processing plant [62]. Lastly, there is no information regarding the quantities that would make chemical recycling a commercially viable process [11].

When considering which recycling method is more viable, Oliveux et al. [26] stated that there is no higher possible value in the outcome of thermal and chemical processes when compared to mechanical recycling and co-processing. For these reasons, those processes are the only economically feasible options [36]. Nevertheless, the viability of the remaining processes depends on applying the recycled material in solutions with higher economical value toward a competitive and profitable process.

## 4. Recycled Glass Fiber-Reinforced Polymer

There is a comprehensive range of applications for rGFRP to be reused. However, they all depend on the overall economic viability of the process. Despite the cost, a few studies have proven for the feasibility of rGFRP to be integrated in the form of filler or reinforcement.

1. Palmer et al. [60] studied the feasibility of replacing virgin fibers with mechanical rGFRP in DMC compounds. With only 10% (in weight) replacement, there was a reduction in the flexural strength of only 8%, compared to the virgin fibers.
2. Pickering et al. [51] studied the substitution of rGFRP SMC panels into a DMC with no significant change in the flexural or impact properties of samples up to 50% recycled material.
3. Gonçalves et al. [36] incorporated mechanical rGFRP into gypsum. This resulted in an increase in the ultimate flexural strength, compared to plain gypsum, of 30%, with the presence of plastic deformation, previously nonexistent.
4. Rahimizadeh et al. [40] used rGFRP from wind blades for reinforcement for fused filament fabrication. The results demonstrated an improvement of approximately 16% in the elastic modulus and an increase in the ultimate flexural strength of 10%, compared to commercially pure PLA filament.
5. Mastali et al. [63] incorporated rGFRP into self-compacting concrete. Using 1.25% (in volume), the following results were observed: 48% increase in the compressive strength, 59% increase in the ultimate flexural strength, and 38% increase in the ultimate crack resistance.
6. Beauson et al. [39] incorporated mechanical rGFRP into the production of chopped stand mats (CSMs), a polyester resin composite. The results showed a significant reduction in strain at which failure occurred, from 1.2% to 1.8% reported in the CSM literature, to 0.3–0.6%.
7. Ribeiro et al. [1,64] have successfully shredded leftovers from the pultrusion profile manufacturing process and studied the effect of incorporating the granulates into polymer mortars on the flexural and compressive strengths, which resulted in an increase in both properties by 13% and 16%, respectively.

R&D projects founded by governmental agencies such as FiberEuse, Dreamwind, and REACT, among others, have worked on the efficiency of recycling technology, designing guidelines for recycled composite reuse improvement and reaching out to stakeholders bridging science and end-consumers in order to guarantee a successful transition to a circular and green economy [21,25].

The results of the previously mentioned authors support the statement that rGFRP can be recycled. Novel engineering materials with appealing mechanical properties are slowly being developed into products such as manhole covers, sound-isolating panels, gypsum boards, construction polymer mortars, molding compounds, pultrusion profiles, car panels, and street furniture [1,25].

### Alternatives to Recycling Processes

The end of usage of a component does not necessarily mean the end of its useful life, as it can be used again as a new refurbished product. The main objective of reusing FRP parts as secondary materials in new products is to maximize the original properties and keep their economic value as high as possible. In addition, the energy required and the costs associated with reutilization processes are significantly lower than those required in recycling processes.

**Figure 16:**
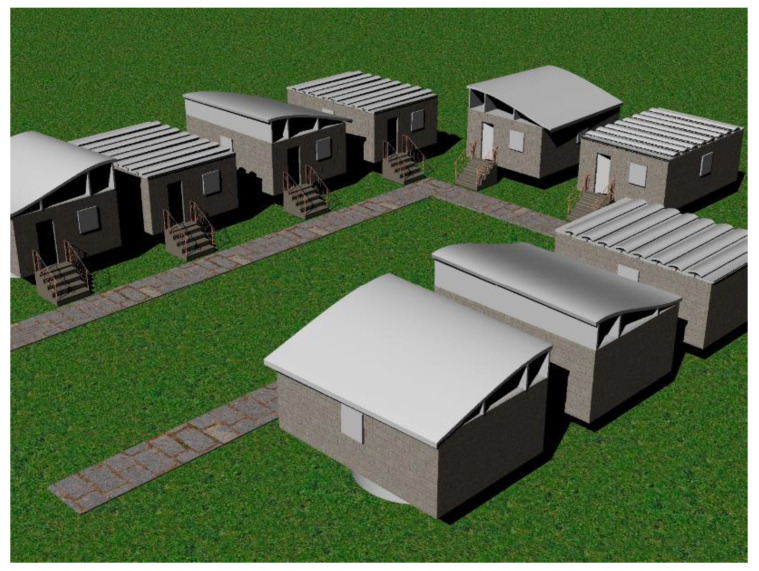
Conceptual housing community [66].

Several studies have been conducted by WindEurope [24] to demonstrate that it is possible to refurbish wind turbine blades into bridges, bike sheds, or street furniture. More recently, a pedestrian bridge made using recycled wind turbine blades was installed in Ireland with great success; thus, public opinion is still to come regarding its appearance [65]. Bank et al. [66] took the concept of refurbishing to building houses with the remains of wind turbine components. The conceptual housing is depicted in Figure 16, where the metal parts of the tower are used to elevate the house from the ground, and the blades are used for the roof in an interlocking configuration.

While such solutions may be appealing, they are not yet viable solutions for the amount of material expected to come to end of life in the coming years. In addition, the components used in these concepts will also come to their end of life again and will, therefore, eventually need to be recycled. The authors of this review believe that the ideal solution may be to redesign the production of the components considering the repurposing of the materials, with multiple uses before recycling, thereby maximizing value retainment.

## 5. Future Perspectives

The future perspectives for the recycling of glass fibers are promising as the scientific community is embracing the development of new hybrid processes with the potential to produce fibers that have added economic value. Therefore, the development of a product composed of such reused material can be brought to the market. However, the success of these technologies is dependent on their industrial scalability and capability to produce large amounts of recycled material in a fast and economical way. Moreover, a key factor will also be the market acceptance of new products, which is now slowly changing to a more sustainable approach due to increasing oil and overall material prices [36].

Although previous attempts to commercialize products containing rGFRP have failed due to a low flow of available recycled material and inherent costs, the trend is for quantities of material to be recycled to increase; thus, the industry must adapt to the development of products containing rGFRP, such as molding compounds, paving blocks, railway sleepers, gypsum wall panels, manhole covers, valve chambers, cement floor screeds, flat sheets for signage, urban furniture, noise-insulating panels, and level crossing panels [1,24,67,68,69].

Nevertheless, the chain of success of the overall process is dependent on the development of clear and specific recycling waste management paths, as well as strong policy and governmental investment promoting and supporting these recycling efforts while banning landfill and residue exportation.

## 6. Conclusions

Overall, there may be successful ways to establish recycling methodologies for glass fiber that allow their use in new applications. However, there is still more to discover and investigate with respect to how the recycling process parameters being used affect the recycled fiber quality and, therefore, the final product. Before proceeding to the development of methods to improve the overall fiber quality, numerical methods for calculating energy demand should also be developed.

It will be interesting to see the development of GFRP recycling technologies on a fully commercialized economic basis in the upcoming years, as the needs to recycle increase and legislation becomes more restrictive. The production of new products containing recycled material will lead to a closed-loop process in which the depreciation of composite materials will decrease as markets seek the use of these materials. Nevertheless, product demand is directly linked to the development path via waste recovery and recycling. This change will happen, whether motivated by economics, market valorization, or environmental legislation. Regardless, the scientific community has a significant responsibility in achieving a sustainable recycling supply chain toward a circular economy and green solution.

The authors of the present review believe that efforts from both academia and industry should be focused on the development of more sustainable recycling processes and methodologies, evaluating the feasibility of development hybrid processes to ensure higher productivity and lower associated costs, developing processing routes that can create graded byproducts targeting specific applications, developing applications where the byproducts of the recycling (other than the recycled fibers) can also be reused, and ensuring the energetic sustainability of the processes devoted to these recycling efforts.

## Figures and Tables

**Table 1:** Overview of process features for recycling of thermoset matrix composites.

Recycling Method	Description	Advantages/Disadvantages	Challenges	Status of the Technology	References
Mechanical	Downsize of material waste into smaller fractions with milling or grinding machines	+ Cost-effective + Low environmental impact + On-site processing − Decrease in fiber mechanical properties − Low market value	Energy evaluation of the process has not been properly addressed Only short fibers and fillers can be recovered	Commercial operations ongoing	[1,4,38,39,40]
Combustion	Integration of the material through co-processing within other materials	+ Highly efficient + Can process large volumes of material + Reduced emissions of cement manufacturing process − No material recovery − Potentially hazardous dust	Increasing gate fees may compromise this route		[11,14,27,41,42]
Chemical	Dissolution of the composite matrix with use of solvents like water, alcohols, or acids	+ Fibers can be recovered with high strength retention + Monomers can be recovered − High energy consumption − Environmentally hazardous	Sizing selection required Scalability development Inherent emissions of the process	Only laboratory-scale Hindered by the fiber market value	[11,26,36,43,44]
Pyrolysis	Decomposition of the materials organic part in an inert high temperature atmosphere	+ Recovered gas or oil can be used as energy for self-sustainability of the process + Low CO_2_ emissions − Fibers with char contamination promote further strength loss upon removal − Long processing time − Only feasible for large quantities			[17,43,45,46,47,48]
Fluidized bed	Hot stream of air is used to decompose the matrix in a silica sand bad, leaving fillers and fibers embedded	+ Contaminated materials can be processed without preprocessing + Recovery of energy or potential precursor chemicals − Higher degradation of the fibers than solvolysis or pyrolysis			[17,42,49,50,51]
